# Dermatosparaxis in two Limousin calves

**DOI:** 10.1186/s13620-016-0074-5

**Published:** 2016-10-18

**Authors:** Catherine I. Carty, Alison M. Lee, Nathan A. E. Wienandt, Edward L. Stevens, Derron A. Alves, John A. Browne, Jill Bryan, Eoin G. Ryan, Joseph P. Cassidy

**Affiliations:** 1 School of Veterinary Medicine, Veterinary Sciences Centre, University College Dublin, Belfield, Dublin 4, Ireland; 2 Joint Pathology Center, 606 Stephen Sitter Avenue, Silver Spring, MD 20910 USA

**Keywords:** Dermatosparaxis, Genetic, Limousin, Bovine, Skin

## Abstract

**Background:**

An unusual presentation of skin disease was identified in two related neonatal Pedigree Limousin calves presented to University Veterinary Hospital, University College Dublin, following detailed post mortem examination a diagnosis of dermatosparaxis was made. Dermatosparaxis in animals or Ehlers Danlos Syndrome, which is the analogous condition seen in humans, is a connective tissue disorder characterised by extreme skin fragility. To the authors’ knowledge this is the first report of such a diagnosis in the Limousin breed and the features of this lethal phenotype were severe in comparison to previous reports of the condition.

**Case presentation:**

Two calves, which were full siblings, a pedigree Limousin bull (Calf A) and pedigree Limousin heifer (Calf B) were examined clinically after presenting collapsed since birth, both had grossly abnormal skin with multiple skin fissures visible and both calves were subsequently euthanised. Both calves underwent gross post mortem examination, after which histological samples were reviewed and electron microscopical examination of selected skin samples was carried out. Histological features of dysplastic dermal collagen were identified. The diagnosis of dermatosparaxis in the Limousin breed was confirmed. Genetic testing was conducted to determine if the current cases had the same mutation as has previously been described in Belgian Blue cattle. Some common parentage was traced but genetic testing did not show a similar mutation to that previously described in cattle. The specific genetic cause in this case is unknown.

**Conclusions:**

This is the first report of dermatosparaxis in the Limousin and the presentation of the dermatosparaxis phenotype has some noteworthy features thus further genetic testing is required to pinpoint the causative mutation or other genetic defect. Given the popularity of the breed and the lethal nature of the phenotype in this case it is important to raise awareness of the condition.

## Background

Dermatosparaxis is an inherited connective tissue disorder in animals primarily recognised by extreme fragility of the skin [1, 2]. The human condition Ehlers-Danlos syndrome (EDS) type VIIC (EDS type VII) is due to a similar biochemical defect in post translational modification of collagen [3]. Although the two skin conditions have analogous pathogeneses, there are marked phenotypic differences. In humans clinical signs include joint laxity with less skin hyperextensibility and fragility than is seen in affected animals [4]. There are marked species and intra-species differences in the expression of the phenotype and the disease has been more aptly described as an Ehlers Danlos Like condition in animals [5]. The condition results from the absence of activity of an enzyme (procollagen I N-proteinase (pNPI)) which normally excises the N-propeptide of type I and type II procollagens resulting in formation of mature collagen. When this enzyme is deficient, there is accumulation of pre-cursor molecules (pro-collagen) that self-assemble into ribbon-like fibrils that fail to provide normal tensile strength to tissues [6]. In normal connective tissue this enzyme excises the amino terminal peptide extension on the precursor molecule to produce collagen. The resulting dysfunctional procollagen in the dermis is poorly structured and loosely arranged, giving rise to hyperextensibility and poor elasticity in the skin of affected animals [1].

Dermatosparaxis has been reported in humans [7], cattle [2], sheep [8], cats [9], horses [10], dogs [11], mink [12], rabbit [5] and buffalo [13]. Cattle breeds affected include the Belgian Blue, Charolais, Hereford, Holstein, Simmental and crossbred cattle [14, 15]. There are no reports of the condition in the Limousin breed.

An autosomal recessive mode of inheritance has been described in Belgian Blue cattle associated with a deletion or single nucleotide substitution that results in a premature stop codon in the “ADAM metalloproteinase with thrombospondin type 1 motif 2” (ADAMTS2) gene. This leads to abnormal processing of procollagen 1 [16]. The recessive nature of the gene was highlighted by the fact that the disease first occurred after inbreedings [2]. However, in a herd of Drakensberger cattle in South Africa where dermatosparaxis occurred, this known mutation was not found [17]. Furthermore, other cases of the condition in other cattle breeds have not been subject to detailed genetic investigation [18].

## Case presentation

**Fig. 1:**
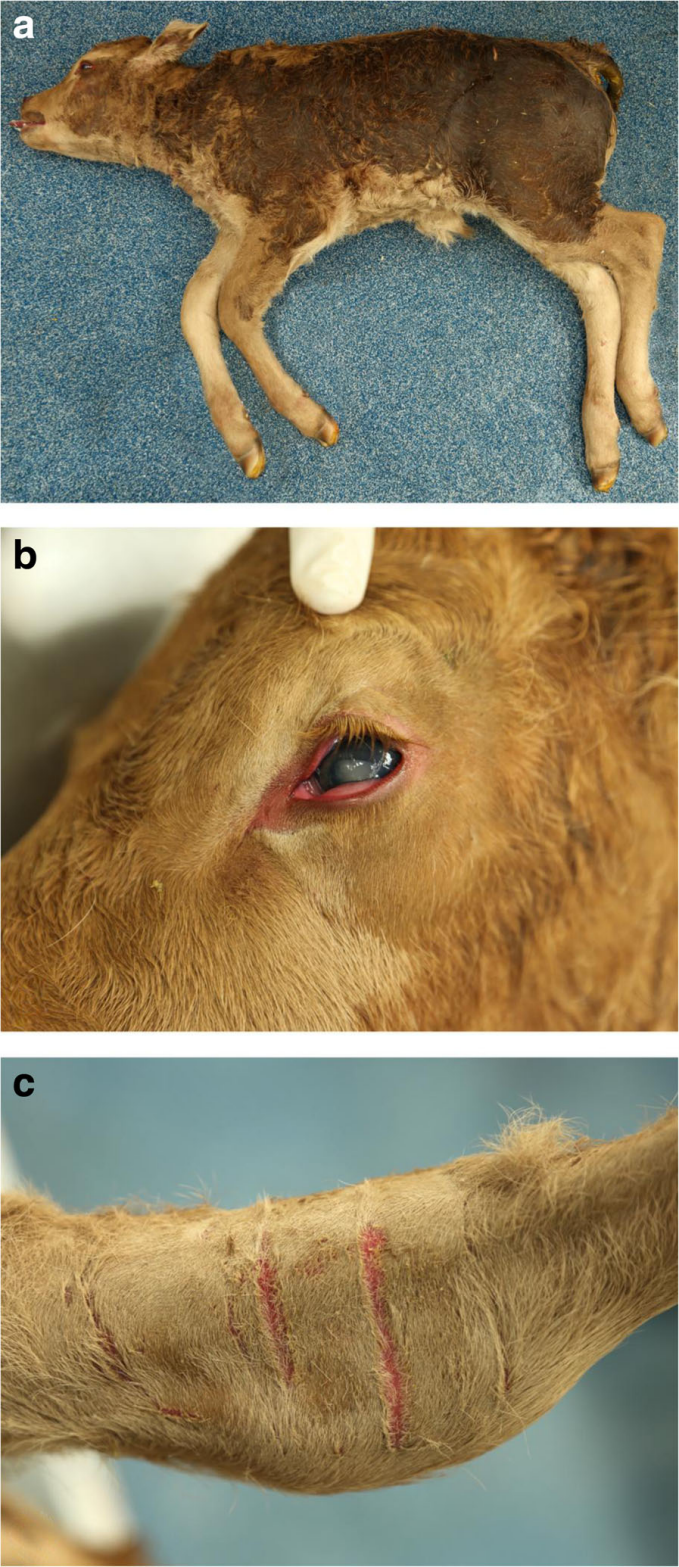
Photographs of Calf A: Picture **a** illustrating diffuse hyperhydration of the skin, Picture **b** “almond” shaped eyes and clouding of lens, Picture **c** linear fissures in the skin

In December 2015 two Pedigree Limousin calves (1 male and 1 female) were presented to the University Veterinary Hospital (UVH) at University College Dublin (UCD). Both were full siblings born to recipient heifers from implantation via embryo transfer. The two calves presented two days apart. The first (Calf A) was a 12-h-old bull calf born unassisted but from birth remained in lateral recumbency and was unable to maintain a normal posture even when assisted. The calf was non-responsive and appeared grossly abnormal, as the skin over the thorax and abdomen was moist (Fig. 1a). The orbital fissures were almond shaped with bilateral clouding of the cornea (Fig. 1b). The calf had thick folds of cervical skin and, on closer inspection, had multiple small fissures in the skin over large parts of the body, particularly over joints and the medial aspect of the hindlimbs (Fig. 1c). Whilst examining the skin more cracks appeared even following gentle manipulation. Additionally this calf displayed involuntary muscle tremors that had been occurring since birth. The calf was euthanised and submitted for post mortem examination. Two days later a second Pedigree Limousin calf (Calf B) presented to the UVH, a female with similar clinical features as Calf A. In addition, Calf B had a ventromedial strabismus bilaterally and pale mucous membranes. This animal was also euthanised and submitted for post mortem examination. Both calves were euthanised using intravenous pentobarbital sodium (Release, 5 ml/10KG BW).

Post-mortem examination revealed the skin of Calf A to be diffusely moderately thickened, with linear fissures noted over the lateral neck, dorsum, and on the flexor surface of the limb joints, axillae, and groin. Occasionally the underlying dermis was exposed. The skin was diffusely damp; the oral mucous membranes were red. Bilaterally, the second incisors were small and subgingival. Calf B had similarly thickened, damp skin, with loose fragments of crusty skin in the regions where the fissures were seen in Calf A. Internal organs of both calves appeared normal; however, there were focally-extensive haemorrhages in the abomasal mucosa of Calf A, with similar, smaller, lesions in Calf B. In both animals the abomasum contained large amounts of clotted milk, but the remainder of the gastrointestinal tract were empty. In addition, there was a small focus of tortuous, dilated blood vessels on the diaphragmatic surface of the spleen of Calf A. All limb joints of Calf B contained a small amount of serosanguinous fluid.

Multiple tissues were sampled from the carcasses for histopathologic processing. Briefly, tissues were fixed in 10 % formalin, dehydrated in alcohol, embedded with wax, cut into 5 μm sections, and mounted on glass slides. All tissues were stained with haematoxylin and eosin (H&E). A selection of skin samples were also stained with periodic acid schiff (PAS), Masson’s Trichrome, and Alcian blue using routine protocols. The Alcian blue stain was employed to look for acid polysaccharides which are found in the ground substance of skin, there was no increase in ground substance in these cases. Tissues were examined using light microscopy by pathologists at UVH UCD and the Joint Pathology Centre, Maryland, U.S.A.

**Fig. 2:**
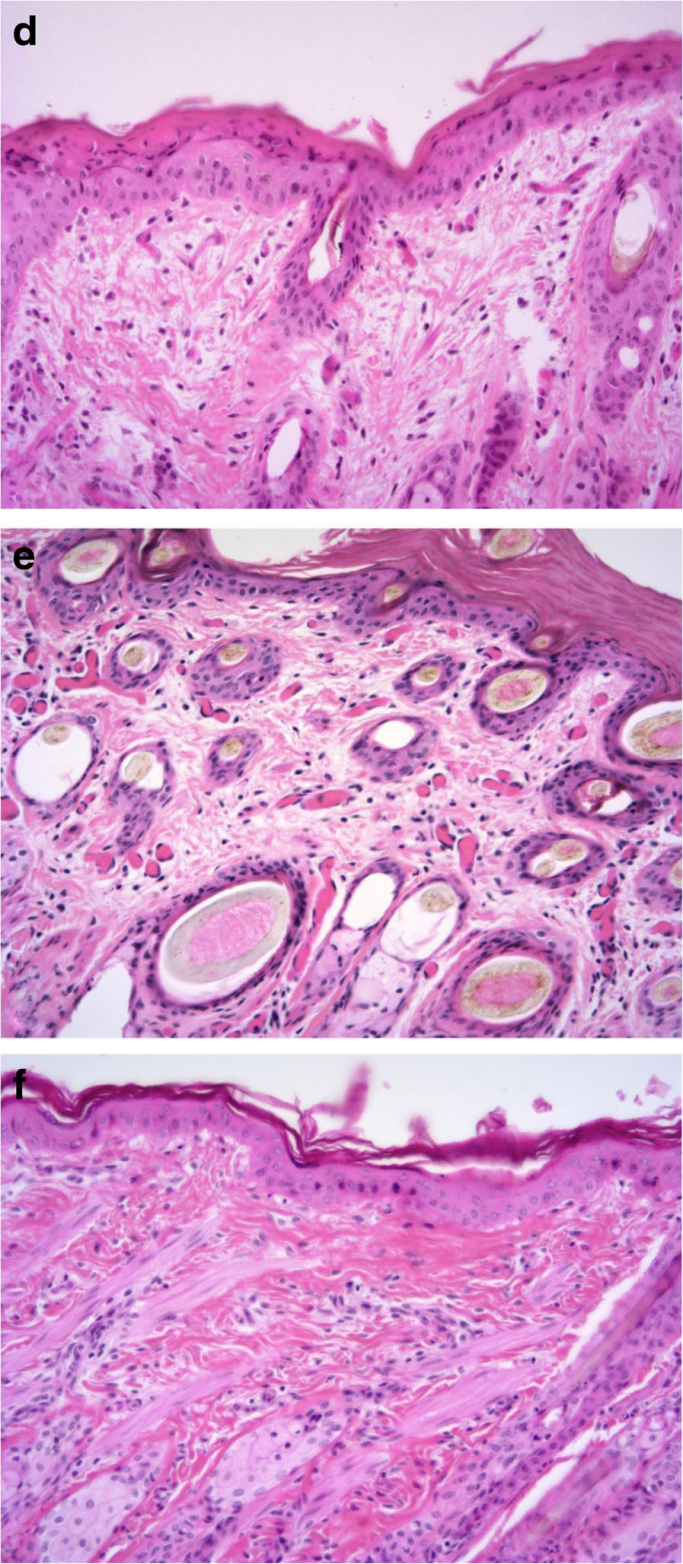
**d & e** Calves A and B, respectively. Dermal collagen fibres are short, fine, fibrillated, fragmented, irregular, and present at a low density. There is mild hyperkeratosis and neutrophilic dermatitis. **f** Control calf. Collagen fibres are longer, thicker, densely packed, and regularly-oriented. H&E, 20x

**Fig. 3:**
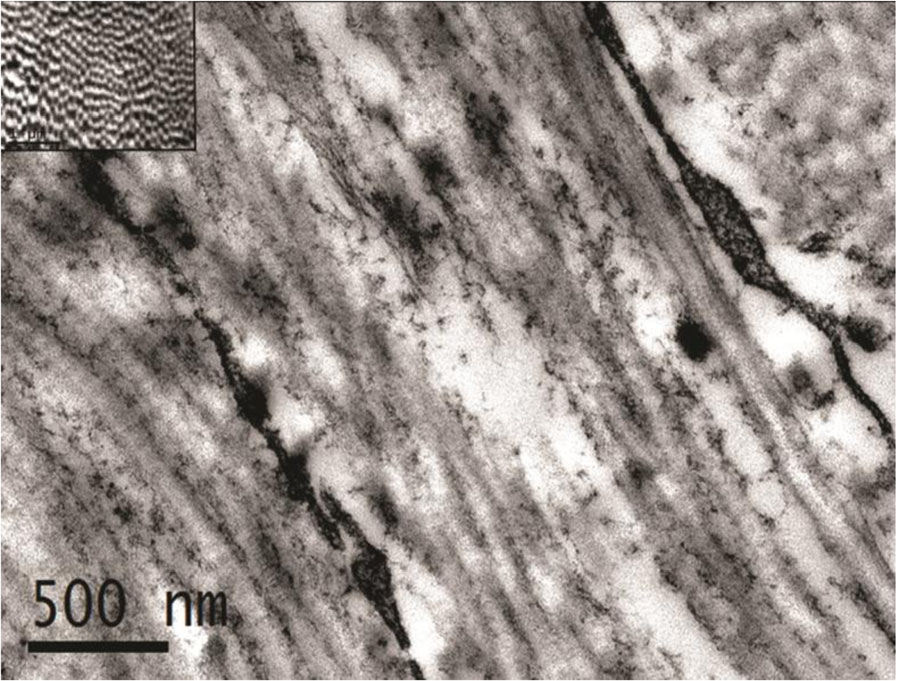
Calf B: Electron Microscopy image of abnormal collagen fibrils. Longitudinal section of irregular, non-parallel and tangled, loosely woven, and occasionally fragmented fibrils with loss of their characteristic periodicity and banding pattern. Inset: Collagen fibrils from an aged matched control calf

Histologically, the skin of Calves A and B exhibited a marked diffuse reduction in the density and number of mature collagen fibres, preferentially affecting the superficial dermis. The remaining fibres were short, fine, pale, fibrillated and fragmented. Trichrome staining by the Masson’s method further highlighted these abnormalities, confirming the dysplastic nature of the collagen. Collagen fibres of the deep dermis appeared relatively spared. Calf B exhibited similar, but less severe, changes (Figs. 2d; e). Ultrastructurally, abnormal dermal collagen fibres of the haired skin of the back consisted of irregular fibrils arranged in loosely woven, twisted flat, and/or helical ribbons with irregularly spaced or lost transverse striations (Fig. 3).

Based on the clinical presentation and the characteristic histological and transmission electron microscopy findings, a diagnosis of dermatosparaxis was made. Given the previously characterised gene mutation reported in Belgian Blue cattle [6], skin samples were examined to discern if the same mutation was responsible in the current case. 5 animals were tested, Calf A, Calf B and three healthy controls (Holstein-Friesian calves). DNA was extracted from fresh frozen skin tissue using the Qiagen DNeasy Blood & Tissue Kit as per the manufacturer’s instructions; DNA quantity was assessed using the NanodropTM spectrophotometer. A primer pair as previously described [17] was used to detect a 17 bp deletion within the coding region of the ADAMTS2 gene;

- Forward Primer 5’- CACCCGCGTGGAGCCCCTGCT-3’
- Reverse Primer 5’- CCAGCCCATCGCAGTTGCTGAG-3’

Ten nanogram of DNA was used for each PCR, reactions were performed in 25 μl and contained 17.5 μl water, 1 AmpliTaq Gold buffer, 2 μl primer mix, 1.5 mM MgCl2, 0.5 μl dNTP mix and 1U of AmpliTaq Gold. The thermal cycling conditions consisted of an initial activation step of 95 °C for 5 min followed by 35 cycles of 94 °C for 30 s, 56 °C for 45 s and 72 °C for 1 min with a final elongation step of 72 °C for 5 min. PCR products were analysed using the Agilent DNA 1000 chip. The PCR product from all DNA samples, both cases and controls, yielded a single sharp band at the expected size of 101 bp, and no deletions, indicating that the genetic defect in these animals is not the same as described previously [6].

The clinical and pathological features described in these two calves are consistent with a diagnosis of dermatosparaxis [1, 2, 16, 17] and is the first reported case of this disease in this breed. The skin fragility and hyperhidrosis, along with the marked fragmentation and depletion of superficial dermal collagen, are consistent with previous bovine cases [1]. Interestingly, the PCR testing carried out in this case did not find a mutation in the ADAMTS gene described previously in cattle. [6, 15] and was similar to the absence of this mutation in Drackensberger cattle [17] highlighting the fact that a range of mutations can result in a broadly similar disease phenotype [5]. Inbreeding has previously been incriminated as the underlying mode of inheritance in Belgian Blue cattle [2] and in the Charolais breed [18]. In the current case, there is common parentage in the ancestry; the dam’s great grandsire and the sire’s grandsire were the same bull.

## Conclusion

This case report highlights the first case of this inherited skin disease in the Limousin breed. The recessive nature of inheritance previously described may suggest that the genetic anomaly is present in the population in a heterozygous state and thus can be preserved [2]. This case also highlights the potential risk of the widespread and rapid dissemination of genetic diseases associated with embryo transfer. In this case, as the specific genetic defect was not identified and considering the lethal nature of the phenotype observed in these calves, we feel further genetic investigation is warranted in order to identify the causative mutation. This information can then be used to screen for the condition in pedigree Limousin animals used to maintain the genepool. Limousin is a popular breed internationally, and raising awareness of this disease should allow the necessary measures to be put in place to minimise its dissemination.
